# Acoustic features of emotional expression in 5-year-old children with autism spectrum disorder

**DOI:** 10.3389/fpsyt.2025.1444675

**Published:** 2025-07-28

**Authors:** Daichi Okuizumi, Kazunori Terada, Azusa Ishii, Yoshimasa Ohmoto, Hitomi Shimizu, Akira Imamura, Ryoichiro Iwanaga, Hirokazu Kumazaki

**Affiliations:** ^1^ Department of Neuropsychiatry, Graduate School of Biomedical Sciences, Nagasaki University, Nagasaki, Japan; ^2^ Hokusuikai Kinen Hospital, Ibaraki, Japan; ^3^ Faculty of Engineering, Department of Electrical, Electronic, and Computer Engineering, Gifu University, Gifu, Japan; ^4^ Faculty of Informatics, Department of Behavior Informatics, Shizuoka University, Shizuoka, Japan; ^5^ Unit of Medical Science, Nagasaki University Graduate School of Biomedical Sciences, Nagasaki, Japan

**Keywords:** autism spectrum disorder, prosody, acoustic feature, F0, emotion

## Abstract

**Background:**

Children with autism spectrum disorders (ASD) exhibit poor prosodic performance, which is associated with their poor language and social skills. Prosody serves important communicative functions not only at grammatical and pragmatic levels but also at the emotional level. This study investigates the acoustic features of emotional expression in children with ASD compared to typically developing (TD) children, within a narrowly defined age cohort restricted to 5-year-old participants.

**Methods:**

Nineteen children with ASD and 19 TD children, aged 5 years, participated in this study. We investigated the differences in the fundamental frequency (f0) ranges in three emotional expression settings (i.e., neutral, liking, and disliking).

**Results:**

The f0 range in the neutral setting was greater in children with ASD than in TD children (*p* = 0.04). There were no significant differences in the f0 range between the three settings in the ASD group (*p* = 0.61). There were significant differences between the neutral and liking settings (*p* < 0.01) and the liking and disliking settings (*p* < 0.01) in the TD group. In the ASD group, a negative correlation was observed between the f0 range in the liking setting and the Social Responsiveness Scale, Second Edition T-score (*p* < 0.01).

**Discussion:**

By focusing on the relationship between acoustic features and emotional expression setting and by restricting the age of participants, our results demonstrate the trend of acoustic features in children with ASD. To deepen the understanding of the relationship between f0 and emotion, future studies investigating prosody in a range of emotional expression settings are needed.

## 1 Introduction

Autism spectrum disorder (ASD) is a developmental disability that causes significant social, communication, and behavioral challenges. The Centers for Disease Control and Prevention (CDC) in the US estimates that one in 36 children has ASD (1). Children with ASD may have difficulty developing language skills and understanding what others say (2), which can limit their opportunities in higher education and employment, resulting in an overall negative impact on their quality of life (3). Additionally, they often struggle to communicate nonverbally through hand gestures, eye contact, facial expressions, and prosody (4).

Prosody is concerned with the suprasegmental features of speech and refers to speech rhythm as well as affective, pragmatic, and syntactic communicative functions (5, 6). Prosody operates at various levels, enabling speakers to construct their speech using expressive language. Children with ASD have some prosodic differences such as atypical intonation (a monotone intonation and robot-like voice), incorrect word stress, speech rhythm differences (too slow or too alert), difficulty using a high or low pitch and controlling intensity, poor resonance (nasalization and pharyngeal resonance) and voice quality (7, 8). Poor prosodic performance may lead to poor language skills in children with ASD (9). Prosodic deficits represent some of the most significant barriers to social integration and acceptance (8), leading to impaired social functioning.

Prosody serves important communicative functions not only at grammatical and pragmatic levels but also at the emotional level (10, 11). The fundamental frequency (f0) is defined as the lowest frequency of the periodic waveform. F0 measures have often been used to identify specific acoustic markers of prosody that differentiate basic emotions (12–15). ASD is associated with impairments in processing one’s own and others’ emotions (16). Grossman et al. (17) reported that the f0 range of individuals with ASD was wider than that of typically developing (TD) individuals when expressing emotions related to gladness, fear, anger, and surprise, in a sample of participants aged 8 to 19 years (17). Hubbard and Trauner (18) reported that the f0 range of individuals with ASD was not significantly different from that of TD individuals in the context of happiness, sadness, and anger, in participants aged 6 to 18 years (18). Hubbard et al. (19) found that the f0 range of individuals with ASD was wider than that of TD individuals in emotional expression contexts involving happiness, sadness, and anger, in participants aged 18 to 50 years (19). However, they reported that the f0 range of individuals with ASD was not significantly different from that of TD individuals for neutral topics. Therefore, the results of previous studies investigating the difference in the f0 range between individuals with ASD and their TD peers according to their emotion are inconsistent. One plausible source of these inconsistencies is the ambiguity of emotion-category labels. The emotion concepts in preschool children are still coarse: between 2 to 5 years of age they tend to group facial expressions mainly by valence and only gradually learn to single out specific categories such as sadness or fear (20). Moreover, constructionist accounts propose that these categories are dynamically assembled from context rather than fixed universals (21, 22). Mapping affect onto the continuous core-affect (valence–arousal) axes may therefore offer a more stable basis for comparing prosody across studies.

The correlation between prosodic features and age is complex, and interactions between them should be considered. It is well known that prosodic features are significantly correlated with speaker age (23). The prosodic features of school-aged children change with age due to factors such as acquiring accents (8). To deepen understanding of the prosodic features, research targeting children before entering elementary school is needed. In our preliminary study (unpublished), which involved children under 4 years of age, understanding the experiment’s explanation proved too difficult, and many participants dropped out. This confounding factor should be minimized by using participants within a narrow age range, restricted to 5 years.

In our preliminary study, we confirmed that 5-year-old children could express emotions of liking and disliking. However, role-playing tasks (i.e., story replay and demonstration tasks) seemed to be difficult for them to complete. By conducting a task that involves showing pictures that provoke the emotions of liking and disliking, it is possible to explore whether emotions can be conveyed through prosody.

McAlpine et al. (24) reported no significant differences in the production of rate, loudness, or pitch between children with ASD and those with TD aged between 24 and 68 months. However, the ASD group exhibited atypical stress patterns significantly more often, such as misplaced stress in multisyllabic words and reduced stress. Yoshimatsu and Umino (25) found that children with ASD scored lower on both prosody comprehension and prosody expression tests compared to typically developing 5-year-old controls. These findings suggest that, among children with ASD around the age of five, prosodic variability and atypicalities are frequently observed.

In this study, we investigated the f0 of children with ASD compared to their TD peers, restricting the sample to 5-year-olds, across different emotional expression settings (i.e., neutral, liking, and disliking). We predicted that our results would reflect a primary difference in the acoustic features of emotional expression in children with ASD.

## 2 Materials and methods

### 2.1 Participants

This study was approved by Hokusuikai Kinen Hospital Institutional Review Board (No. 081). The legal guardians of the participants provided written informed consent, and the participants provided assent to participate in this study. All procedures involving human participants were conducted in accordance with the ethical standards of the institutional and/or national research committee and the 1964 Declaration of Helsinki and its later amendments or comparable ethical standards. After receiving a complete explanation of the study, all participants and their guardians agreed to participate. All participants and their guardians provided written informed consent. The inclusion criteria for the ASD group were as follows: they had a diagnosis of ASD based on the Diagnostic and Statistical Manual of Mental Disorders, Fifth Edition (DSM-5) by a supervising study psychiatrist (26); they were 5 years of age; and their IQ scores were 70 or higher. During enrollment, the diagnoses of all participants were confirmed by a psychiatrist with more than 15 years of experience in ASD using standardized criteria derived from the Diagnostic Interview for Social and Communication Disorders (DISCO), which has demonstrated good psychometric properties (27, 28).

Children with TD were recruited from a public offering. The inclusion criteria for the TD group were: children had to be 5 years of age, have a Social Responsiveness Scale, Second Edition (SRS-2) T-score of 59 or lower, and attend a mainstream preschool with no evidence of intellectual impairment. A total of 19 children with ASD and 19 TD participants were included in this study.

Parents of children in both groups completed the SRS-2 (1) to screen for clinically significant autistic symptoms. Higher scores on the SRS-2 indicate a higher degree of autistic traits. Raw SRS-2 scores were converted to T-scores (with a mean of 50 and a standard deviation of 10) for each sex. We classified the data as TD based on a cutoff value “59” according to the previous study (29).

The participants also completed the Social Communication Questionnaire (SCQ) (30). The SCQ is frequently used as a screening tool in ASD research. It was designed as a questionnaire version of the Autism Diagnostic Interview-Revised (ADI-R; (31)), the gold-standard developmental history measure widely used in research and clinical practice. In this study, we did not set a cutoff based on the SCQ score and only used the SRS-2 to gather children with TD.

Full-scale IQ scores were obtained using the Wechsler Preschool and Primary Scale of Intelligence-Third Edition (WPPSI-III).

There were significant differences in the SCQ total scores (*p* < 0.01) and SRS-2 T-scores (*p* < 0.01) between children with ASD and their TD peers. Details of the demographic data are presented in **Table 1**.

**Table 1:** Demographic data of participants.

	ASD (*n* = 19)	TD (*n* = 19)	
	Mean (SD)	Mean (SD)	*p*
Age (months)	64.79 (3.81)	66.95 (2.50)	0.06
Sex ratio (M: F)	13:6	13:6	
SCQ total score	10.16 (4.60)	2.21 (1.51)	<0.01**
SRS-2 T-scores	71.79 (11.21)	44.42 (5.22)	<0.01**
IQ	83.79 (9.10)	N/A	N/A

SD, standard deviation; ASD, autism spectrum disorder; TD, typical development; SCQ, Social Communication Questionnaire; SRS-2, Social Responsiveness Scale-Second edition.

**p < 0.01.

### 2.2 Materials

In this study, we categorized the participants’ speech into three emotional expression settings: neutral, liking, and disliking. These settings were selected because they occupied distinct regions along the valence-arousal and valence dimensions (32). Utterance in the liking setting evoked high arousal and positive valence, whereas utterance in the disliking setting evoked low arousal and negative valence.

**Figure 1:**
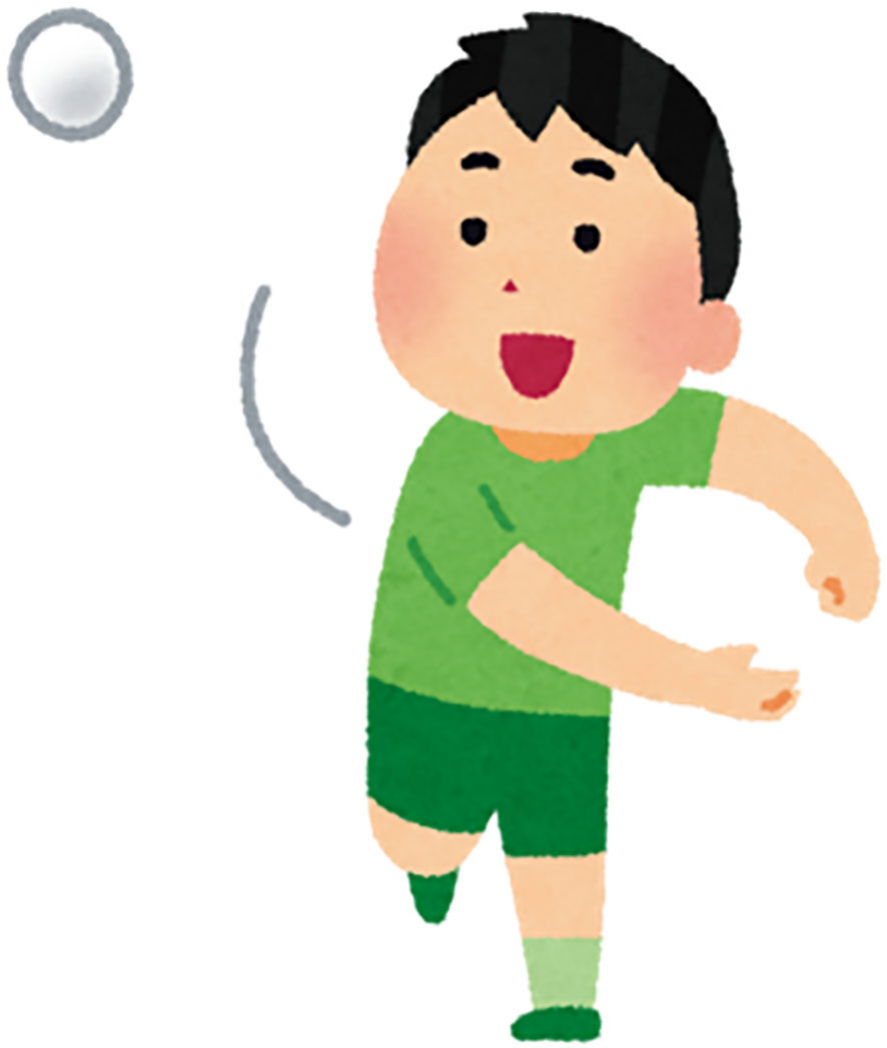
Example of action pictures.

**Figure 2:**
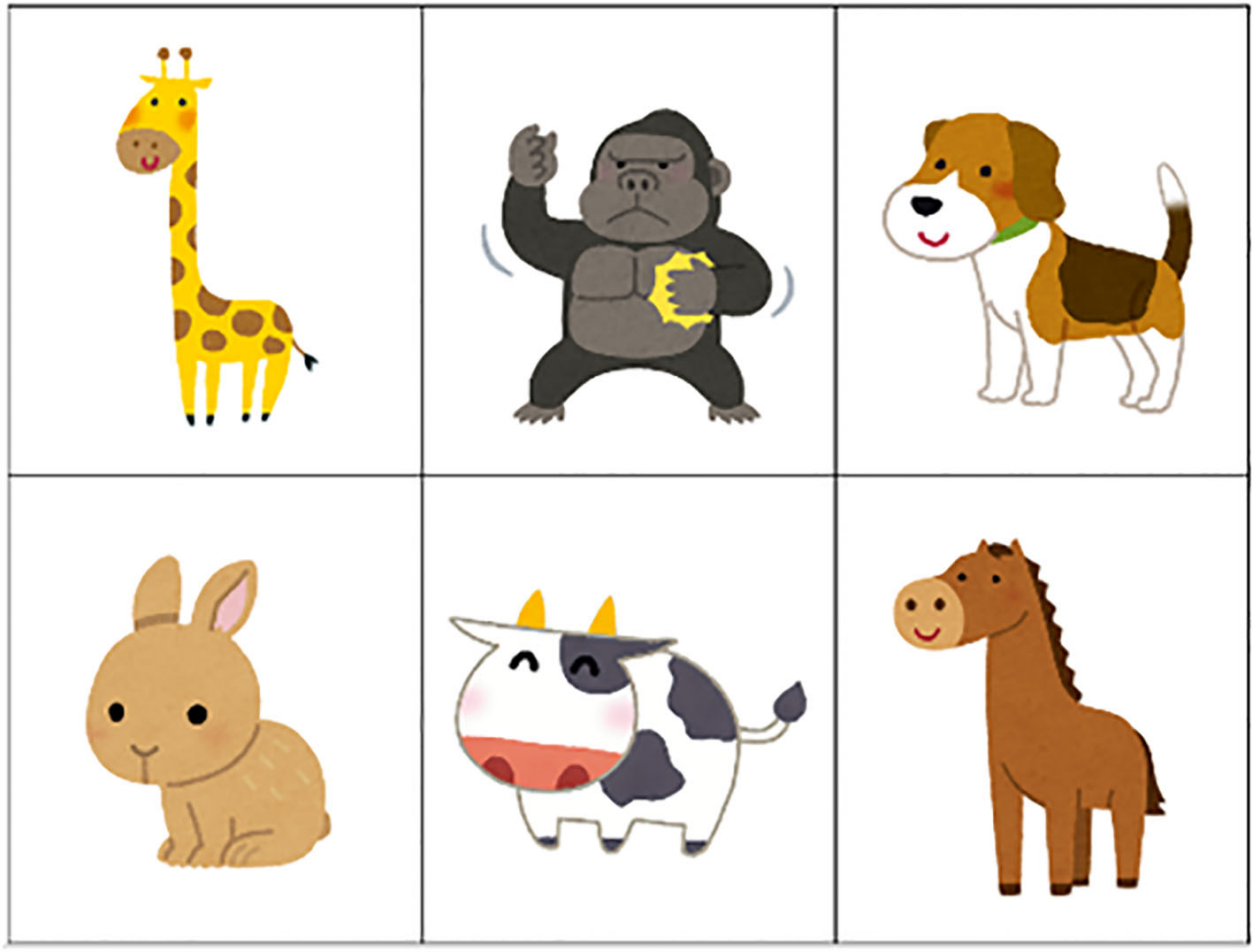
Example of category pictures.

Teaching materials were created using vocabulary acquired before 3 years of age, based on the MacArthur-Bates Communicative Development Inventories (MB-CDIs) (33), which evaluate language development based on parent reports. The teaching materials included actions and pictures of the categories. The pictures were displayed on a tablet. We used 10 action-picture cards (throwing a ball, eating rice, swimming in the pool, walking on the road, waking up in the morning, cutting a tree, sitting on a chair, kicking the ball, drinking water, and putting on clothes). Examples of action pictures are illustrated in **Figure 1**. We also used 14 categories of picture cards (animals, sea creatures, 4-wheeled vehicles, vehicles, indoor toys, outdoor toys, food, vegetables, fruits, teenagers, colors, outings, occupations, and characters). In each picture card category, six nouns corresponded to one category. An example category image is depicted in **Figure 2**.

### 2.3 Procedure

Speech recordings in the three emotional expression settings were conducted in the following order: neutral, liking, and then disliking. Examiners of emotional expressions were blinded to the diagnosis group.

In the neutral setting, 10 action-picture cards were displayed one after the other, and participants were instructed to verbally name each action (e.g., “throw a ball”). After displaying all 10 action-picture cards, participants took a 1-min break before repeating the exercise. Each picture card was displayed, and the examiners asked the participants to name the object shown. Since the examiner did not utter the name of the object, the participants were unable to imitate their utterances directly. If the participants gave an incorrect response or did not answer, the data were removed from the analysis, and the examiners displayed the next action-picture card.

In the liking and disliking settings, 14 category picture cards were displayed individually. Participants were encouraged to select a card and express their liking as “I like giraffes.” Subsequently, they were encouraged to select another card and express their dislike as “I don’t like gorillas.” If the participants provided an incorrect response or did not answer, the examiners displayed the next picture card. The participants checked their expressed emotions using a 5-point Likert scale after completing all the sentence tests. The recording equipment consisted of a microphone (the Shure MV7) and an audio capture system (the TASCAM PortaCapture X8). The microphone was maintained at a constant distance from the participants during recording. The recording parameters included a 48-kHz sampling frequency and 24-bit quantization.

### 2.4 Criteria for utterance selection

To obtain relatively comparable speech samples from participants’ utterances, only complete utterances containing both a noun and a verb were included in the analysis, provided they did not meet any of the exclusion criteria. Utterances were excluded if they were questions, fillers, repetitive speech, consisted only of a noun or a verb, were interrupted by the examiner, were unintelligible, were directed toward someone else in the room and unrelated to the task, or were abandoned. Only complete utterances containing more than two words were considered. In the neutral setting, action utterances (e.g., “throw a ball”) were selected, whereas in the liking and disliking settings, preference utterances (e.g., “I like giraffes”) were chosen. These exclusion criteria were applied to ensure consistency in utterance length and type across participants.

### 2.5 Measurement

The audio data for analysis consisted of utterances in three settings. Using sound editing software (free software, Praat version 6.2.14), the sampling frequency of the recorded data was converted to 24 kHz, and the audio data were isolated for each sentence. We checked all audio data for artifacts that might interfere with accurate pitch analysis (e.g., background noise, coughs, or artifacts from glottal stops) and removed them. For each sentence, we extracted f0 (in Hz) with a timestep of 0.01 seconds. The f0 range, which measures the extent to which an individual’s pitch varies during speech, was calculated by subtracting the minimum f0 value from the maximum f0 value obtained from each sentence. First, the f0 range of each sentence was calculated for each emotional expression setting. The f0 range in each setting was subsequently averaged across all sentences.

### 2.6 Data analysis

Statistical analyses were performed using IBM SPSS Statistics for Windows, version 24.0 (IBM Corp., Armonk, NY, USA). Differences in the number of speeches between children with ASD and TD were analyzed using Mann–Whitney *U* tests. Kruskal–Wallis tests were used to determine if differences in the f0 range existed in each emotional expression setting (i.e., neutral, liking, disliking) between children with ASD and TD. The Friedman test was used to determine the f0 range in each setting in children with ASD and TD. Spearman’s rank correlation coefficients were used to explore the relationships between the f0 range and the SRS-2 T-score and between the f0 range and their IQ in each setting in children with ASD.

## 3 Results

No significant difference was observed between the number of sentences in children with ASD and TD (neutral setting, *U* = 121.50, *p* = 0.09; liking setting, *U* = 131.00, *p* = 0.15; disliking setting, *U* = 142.00, *p* = 0.27). The details are presented in **Table 2**.

**Table 2:** The number of sentences in children with ASD and TD children in Neutral, Liking, and Disliking settings.

	ASD (*n* = 19)	TD (*n* = 19)	
	Mean (SD)	Mean (SD)	*p*
Neutral setting	7.68 (1.80)	8.63 (1.42)	0.09
Liking setting	11.79 (2.52)	13.05 (1.08)	0.15
Disliking setting	7.32 (4.10)	8.84 (3.50)	0.27

ASD, autism spectrum disorder; TD, typical development; SD, standard deviation.

The f0 range in the neutral setting was greater in children with ASD than TD children (*H* = 4.24, *df* = 1, *p* = 0.04). There were no significant differences in the f0 range across groups for the liking setting (*H* = 0.79, *df* = 1, *p* = 0.37) or disliking setting (*H* = 1.26, *df* = 1, *p* = 0.26) between children with ASD and TD children.

**Figure 3:**
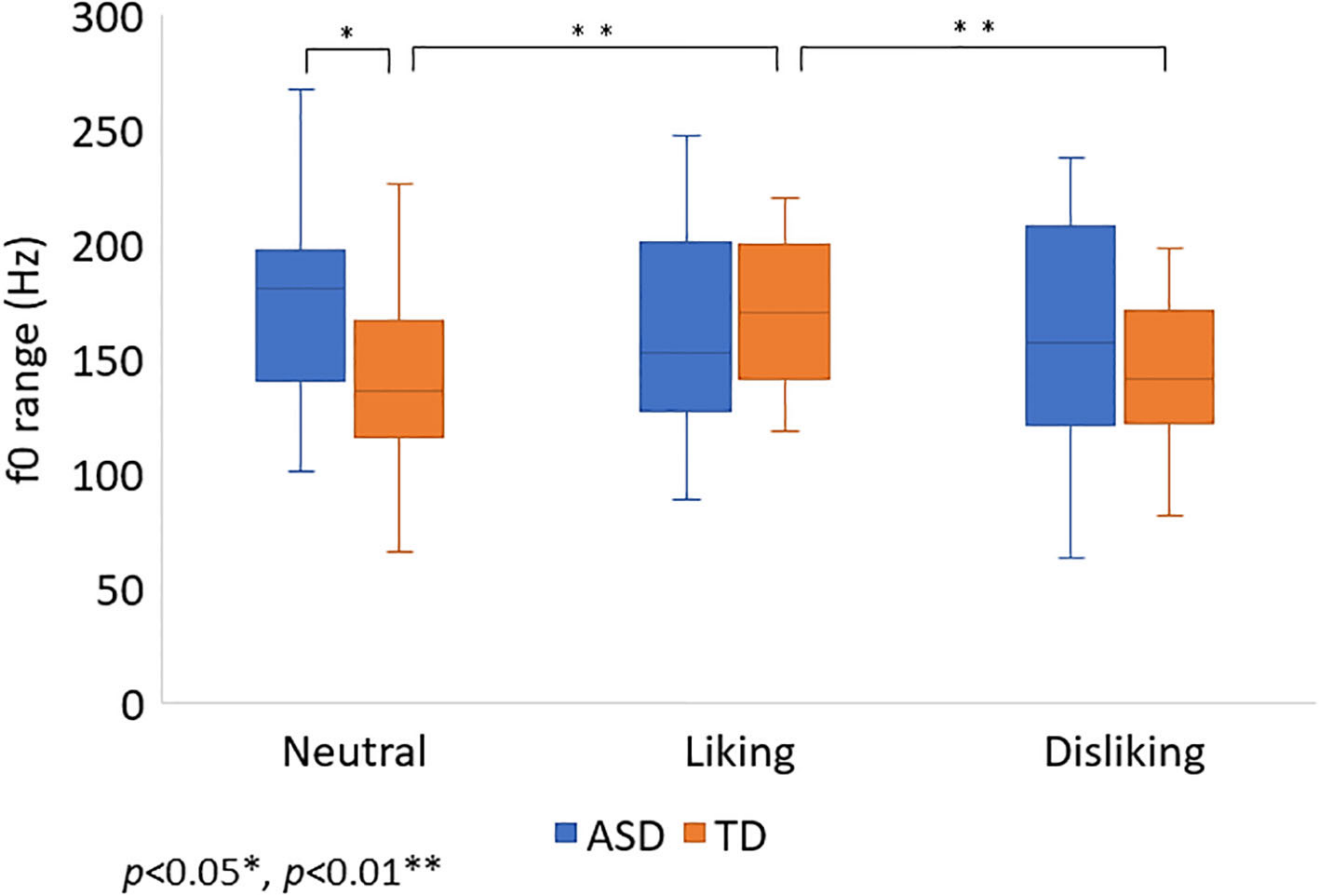
The f0 range in neutral, liking, and disliking settings in children with ASD and TD. The f0 range in neutral, liking, and disliking settings in children with ASD and TD is shown. Error bars indicate ±1 standard error of the mean. In the neutral setting, children with ASD exhibited a significantly wider f0 range than those with TD (*p* = 0.04). Among children with TD, significant differences in the f0 range were observed between the liking and neutral settings (*p* < 0.01), and between the liking and disliking settings (*p* < 0.01).

There were no significant differences in the f0 range between the three settings in the ASD group (*χ²* (2) = 1.00, *p* = 0.61). Bonferroni-corrected *post-hoc* tests revealed no significant differences between the neutral and liking settings (*p* = 0.31), the neutral and disliking settings (*p* = 0.62), or the liking and disliking settings (*p* = 0.62) in the ASD group. There were significant differences in the f0 range between the three settings in the TD group (*χ²* (2) = 13.37, *p* < 0.01). Bonferroni-corrected *post-hoc* tests revealed that the liking setting had a greater f0 range than the neutral context (*p* < 0.01) and the disliking setting (*p* = 0.01) in the TD group. There were no significant differences between the neutral and disliking settings in the TD group (*p* = 0.87). The details are illustrated in **Figure 3**.

In children with ASD, a negative correlation was observed between the f0 range in the liking setting and the SRS-2 T-score (*r* = -0.60, *p* < 0.01). There were no significant differences in the correlations between the SRS-2 T-score and f0 range in the neutral (*r* = -0.17, *p* = 0.49) and disliking settings (*r* = -0.38, *p* = 0.12). The details are presented in **Table 3**. There were no significant differences between IQ and f0 in the neutral (*r* = 0.11, *p* = 0.67), liking (*r* = 0.18, *p* = 0.46), or disliking (*r* = 0.40, *p* = 0.09) settings. The details are presented in **Table 3**.

**Table 3:** The correlations between SRS-2 T-scores and IQ, and f0 range of neutral, liking, and disliking settings.

Item	SRS-2 T-scores	IQ
Neutral setting	-0.17	-0.11
Liking setting	-0.60**	0.18
Disliking setting	-0.38	0.40

SRS-2, Social Responsiveness Scale.

**p < 0.01.

## 4 Discussion

This study investigated the f0 of children with ASD compared to their TD peers, restricting the age range of participants to 5 years, across different emotional expression settings (i.e., neutral, liking, and disliking) within a narrow age range restricted to 5 years. The results revealed that the f0 range in the neutral setting was greater in children with ASD than in TD children. This suggests that in emotionally neutral situations, children with ASD may show greater pitch variation compared to TD children. There were no significant differences in the f0 range between the three settings (i.e., neutral, liking, disliking) in the ASD group, whereas significant differences in the f0 range were observed between the three settings in the TD group. These findings suggest that children with ASD have difficulty varying pitch according to their emotions, whereas children with TD emphasize positive emotions in their speech. In children with ASD, a negative correlation was observed between the f0 range in the liking setting and the SRS-2 T-score, suggesting that increased severity of autistic traits is associated with reduced pitch variation. Our results indicate a trend in the acoustic features of children with ASD.

Previous studies (34–37) have reported that the f0 range in general conversations in individuals with ASD was higher than that in TD individuals, which is consistent with the results of this study, that the f0 range in the neutral setting in children with ASD was greater than that in TD children. No significant differences were observed in the f0 range between the ASD and TD groups in the liking and disliking settings. This finding is consistent with those of Hubbard and Trauner (18), who reported no significant differences in the f0 range between ASD and TD groups aged 6 to 21 years during emotional expression. Conversely, Hubbard et al. (19) reported significant differences in the f0 range between ASD and TD groups aged 21 to 41 years during emotional expression. According to a previous study by Lee et al. (38), f0 variability usually decreases with age, beginning around 10 years of age. A previous meta-analysis (7) suggested that pitch difference between individuals with ASD and those with TD were significant during adulthood compared to other age groups, which may explain the discrepancies between the findings of Hubbard and Trauner (18) and those reported by Hubbard et al. (19). Given these factors, the finding of the present study–that there were no significant differences in the F0 range between the ASD and TD groups in both the liking and disliking settings–is understandable.

Contrary to their TD peers, children with ASD are unable to change pitch in areas where it is usually emphasized and are unable to adjust pitch depending on the communication situation (39). These results may explain why children with TD emphasize positive emotions in their speech, whereas children with ASD have difficulty varying pitch according to their emotions.

Nakai et al. (40) reported that pitch variation in word utterances was negatively correlated with the severity of autistic traits (40). A previous study also found that pitch measures extracted from the ADOS-2 conversational task were significantly negatively correlated with the SRS-2 T-score (41).

By focusing on the relationship between acoustic features and emotional expression settings, and by restricting the age range of participants, our study found a negative correlation between the f0 range in the liking condition and autistic traits, consistent with previous findings (8, 40). Given that the f0 range is associated with emotional expression (12–15) and that emotional expression in favorable settings is linked to social functioning (42), the f0 range in emotional contexts may reflect social dysfunction in children with ASD.

When considered in the context of previous studies (8, 40), our findings support the notion that greater severity of autistic traits is associated with reduced f0 range, even in positive emotional contexts. These results underscore the potential future applications of (i) developing voice-based biomarkers utilizing the f0 range, and (ii) implementing interventions targeting emotional prosody.

Our findings—that children with ASD have difficulty modulating pitch according to emotional context, particularly in the lower f0 range of the liking condition, which is associated with greater autistic traits—can be interpreted within the framework of neurodevelopmental theories of emotional processing and prosody.

Emotional prosody processing is typically associated with right-hemisphere brain regions, including the right superior temporal gyrus and inferior frontal gyrus (43, 44). Atypical development or reduced activation in these regions has been reported in individuals with ASD (45), which may underlie difficulties in modulating vocal pitch to match emotional valence.

Furthermore, theory of mind (ToM)—the ability to infer others’ mental and emotional states—is also implicated in prosodic expression (46). If children with ASD have reduced ToM abilities, they may not only struggle to interpret others’ emotional prosody but may also have difficulty expressing their own emotions vocally in socially appropriate ways.

These findings suggest that a reduced f0 range in children with ASD, even in positive emotional contexts, reflects underlying neurodevelopmental mechanisms affecting both emotional processing and social communication. This highlights the importance of incorporating such theoretical frameworks when developing voice-based biomarkers and interventions targeting emotional prosody.

### 4.1 Limitations

This study has several limitations. First, the sample size was relatively small, and most participants were male. Wehrle et al. (47) described the prosody of men with ASD as more exaggerated than that of women. Furthermore, parents have reported that boys with ASD are more likely to speak with an “unusual tone of voice” than girls (48). Therefore, gender differences in prosody may exist among children with ASD. Second, we did not conduct formal IQ testing in children with TD and instead relied only on the records of a normal preschool performance for those enrolled in this study. However, all the children attended mainstream preschools with no evidence of intellectual impairment. We confirmed that 5-year-old children with average intellectual and verbal competencies could complete the experimental process in our preliminary experiments. These results also demonstrated that IQ was not correlated with the f0 range in children with ASD. Previous longitudinal research (49) suggests that age-appropriate performance in preschool reliably predicts later cognitive functioning, including IQ. Therefore, assuming average IQ in TD children based on typical preschool performance may be justified, although we acknowledge the limitations of inferring cognitive level without formal testing. This limitation should be considered when interpreting between-group comparisons, as individual differences in cognitive ability within the TD group may not be fully accounted for in the present study. Third, since the participants were 5 years old, it was difficult to perform an experiment involving complex procedures. This study focused on the neutral, liking, and disliking settings. It is generally accepted that there are various emotions such as happiness, sadness, anger, disgust, fear, and surprise.

### 4.2 Future research directions

To address these limitations and promote advanced understanding, we highlight the following: First, studies with larger sample sizes, including numerous female participants are required to validate the results. Second, further research assessing IQ in TD children is needed. Third, further studies are needed to investigate f0 in a variety of emotional expression settings in order to advance our understanding of the relationship between f0 and emotion.

## 5 Conclusion

This study identified the acoustic features according to each emotional expression setting in children with ASD compared to TD children. Given the relationship between prosody, language skills, and social dysfunction, focusing on prosodic features screening children whose social function is poor, the significance of focusing on prosody is great. Further studies are needed to investigate f0 in a variety of emotional expression settings, in order to deepen our understanding of the relationship between f0 and emotion.

## Data Availability

The raw data supporting the conclusions of this article will be made available by the authors, without undue reservation.
